# *Sphingobacterium pedocola* sp. nov. a novel halotolerant bacterium isolated from agricultural soil

**DOI:** 10.1007/s10482-021-01623-6

**Published:** 2021-08-06

**Authors:** Ákos Tóth, Ildikó Bata-Vidács, Judit Kosztik, Rózsa Máté, József Kutasi, Erika Tóth, Károly Bóka, András Táncsics, István Nagy, Gábor Kovács, József Kukolya

**Affiliations:** 1 Research Group for Food Biotechnology, Institute of Food Science and Technology, Hungarian University of Agriculture and Life Sciences, Budapest, Hungary; 2 BioFil Microbiological, Biotechnological and Biochemical Ltd, Budapest, Hungary; 3 Department of Microbiology, Eötvös Loránd University, Budapest, Hungary; 4 Department of Plant Anatomy, Eötvös Loránd University, Budapest, Hungary; 5 Regional University Center of Excellence in Environmental Industry, Szent István University, Gödöllő, Hungary; 6 SeqOmics Biotechnology Ltd., Mórahalom, Hungary; 7 Institute of Biochemistry, Biological Research Centre, Eötvös Lorand Research Network, Szeged, Hungary; 8 University of Sopron, Sopron, Hungary

**Keywords:** *Bacteroidetes*, New taxon, *Sphingobacteriaceae*, *Sphingobacteriales*, *Sphingobacterium pedocola*

## Abstract

A Gram-reaction-negative halotolerant bacterial strain, designated Ka21^T^, was isolated from agricultural soil and characterised using a polyphasic approach to determine its taxonomic position. On the basis of 16S rRNA gene sequence analysis, highest similarity was found with *Sphingobacterium alkalisoli* Y3L14^T^ (96.72%). Cells were observed to be aerobic, non-motile rods. The isolate was found to be able to grow between 0 and 10% of NaCl concentration. The assembled genome of strain Ka21^T^ has a total length of 5.2 Mb with a G + C content of 41.0 mol%. According to the genome analysis, Ka21^T^ encodes several glycoside hydrolases that may play a role in the degradation of accumulated plant biomass in the soil. Based on phenotypic characteristics and phylogenetic analysis, it is concluded that strain Ka21^T^ represents a novel species in the *Sphingobacterium* genus for which the name *Sphingobacterium pedocola* sp. nov. is proposed. The type strain of the species is strain Ka21^T^ (= LMG 31575^T^ = NCAIM B.02636^T^).

**Supplementary Information:**

The online version contains supplementary material available at 10.1007/s10482-021-01623-6.

## Introduction

The genus *Sphingobacterium* was proposed by Yabuuchi et al. (1983), and it belongs to the family *Sphingobacteriaceae*, order *Sphingobacteriales*, class *Sphingobacteriia*, phylum ‘*Bacteroidetes*’ (Steyn et al. 1998). At the time of writing, the genus includes 60 validly published species with correct name (https://lpsn.dsmz.de/ June, 2021), the type species is *Sphingobacterium spiritivorum* (Euzéby 1997; Parte et al. 2020). The main characteristics of the genus are Gram-staining negative, catalase and oxidase positive, rod shaped, non-motile and MK-7 as predominant isoprenoid quinone. Most of the *Sphingobacterium* strains have been isolated from soil, rhizosphere or composts. In these environments, the decomposition of dead plant material is mediated by cellulolytic bacteria. The key enzymes in the lignocellulose degradation are the various glycoside hydrolases. Salt-tolerant enzymes may have advantages in many industrial applications (Oren 2010; Yin et al. 2015), particularly for utilisation in nonaqueous media (van den Burg 2003). The domain *Bacteria* contains many types of halophilic and halotolerant microorganisms. Although several classifications have been proposed, the most widely used definitions for these bacteria were formulated by Kushner (1978). Bacteria able to grow in the absence of salt as well as in the presence of relatively high salt concentrations are designated halotolerant (Ventosa et al. 1998). Here, we report a polyphasic taxonomical description of a novel, slightly halotolerant bacterium strain, designated Ka21^T^. The phenotypic, chemotaxonomic and genotypic properties indicate that strain Ka21^T^ represents a novel species within the genus *Sphingobacterium*, for which the name *Sphingobacterium pedocola* sp. nov. is proposed.

## Materials and methods

### Isolation and cultivation

Strain Ka21^T^ was isolated from an agricultural field in the Great Hungarian Plain. Before sampling, maize was harvested from the field. After sampling, the soil particles were homogenised by vortexing and serially diluted with peptone water (9 g peptone, 1 g NaCl, in 1000 ml dH_2_O). 100–100 µl of the third to the fifth member of the dilution series was subsequently spread onto xylan containing agar (1 g NaNO_3_; 1 g K_2_HPO_4_; 3 g NaCl; 0.5 g MgCl_2_; 0.5 g yeast extract; 0.5 g peptone; 3 g xylan; 25 g agar; 1000 ml dH_2_O) and incubated at 10 °C for 5 days. Single colonies on the plates were purified on the same medium. The isolate was routinely maintained on LB medium (DSM medium No. 381, www.dsmz.de) at 28 °C and pH 7.5.

### Physiology and chemotaxonomy

Biomass for chemical and molecular studies was obtained by cultivation in shaker flasks using LB medium at 30 °C for 32 h. Colony morphology of strain Ka21^T^ was tested on LB agar medium by directly observing single colonies. Cell morphology of strain Ka21^T^ was observed by electron microscopy. The Gram reaction was determined with a non-staining method as described by Buck (1982). Oxidase activity was studied with OXI oxidase test strip (Diagnostics s.r.o.). Catalase production was demonstrated by the method of Barrow and Feltham (2004). Growth at different temperatures (from 4 to 50 °C), NaCl tolerance (0–12% w/v) and pH tolerance (pH 4–10, using increments of 0.5 pH units) were determined using LB medium. Acid production from different carbon sources, the assimilation of different substrates and the enzymatic activities of strain Ka21^T^ were investigated with API 50 CH, API 20 NE and API ZYM kits (BioMérieux) according to the manufacturer’s instructions. The API 50 CH and 20 NE tests were read after 24–48 h incubation at 30 °C. Anaerobic and microaerophilic growth was checked on LB medium using the Anaerocult A and C systems (Merck).

Analyses of chemotaxonomic traits were carried out by DSMZ Identification Service (DSMZ, Braunschweig, Germany). The fatty acid profiles of strain Ka21^T^ was analysed on active growing cultures on LB agar. According to the DSMZ Identification Service, fatty acid methyl esters (FAMEs) were obtained following the methods of Miller (1982) and Kuykendall et al. (1988). FAMEs were separated by gas chromatography, detected by a flame ionisation detector using Sherlock Microbial Identification System (MIS) (MIDI, Microbial ID, Newark, DE 19711 U.S.A.) and identified by using the TSBA6 6.10 database of the Microbial Identification System. Summed feature components were identified thereafter by GC/MS.

The respiratory quinones were extracted from freeze dried material and purified by a silica-based solid phase extraction. Purified samples were further analysed by HPLC and UHPLC-ESI-qTOF system (Tindall 1990a, b; dsmz.de]. Polar lipids were studied according to Tindall et al. (Tindall 1990a, b; Tindall et al. 2007; dsmz.de).

### Genome features

DNA was extracted from Ka21^T^ liquid culture grown in LB medium. Genomic DNA isolation and 16S rRNA gene amplification were performed according to Tóth et al. (2017). The genome of strain Ka21^T^ was sequenced with Illumina MiSeq sequencing technology as described previously (Szuroczki et al. 2019). Genome assembly was performed by SPAdes v. 3.9.1; CLC NGS Cell v. 11.0. Genome completeness and contamination values were examined by TypeMet tool of MiGA server (http://microbial-genomes.org/) (Rodriguez et al. 2018). Annotation of the genome was performed by NCBI Prokaryotic Genome Annotation Pipeline v4.4 with Best-placed reference protein set and GeneMarkS + methods (Tatusova et al. 2016; O’Leary et al. 2016) and Rapid Annotation using Subsystem Technology server v. 2.0 (RAST; https://rast.nmpdr.org) (Aziz et al. 2008).

The anti-SMASH server was used to identify the secondary metabolite biosynthesis gene clusters (Blin et al. 2019). Comparative genome analysis for *Sphingobacterium pedocola* Ka21^T^, *Sphingobacterium alkalisoli* Y3L14^T^ (Xu et al. 2017), *Sphingobacterium olei* HAL-9^T^ (Liu et al. 2020) and *Sphingobacterium composti* DSM 18850^T^ (Yoo et al. 2007, later homonym of *Sphingobacterium composti* T5-12^T^ (Ten et al. 2006)) was performed by OrthoVenn2 webserver (https://orthovenn2.bioinfotoolkits.net/) (Xu et al. 2019). Methabolic pathways were analysed using the MicroScope platform (Vallenet et al. 2009).

### Phylogeny

The partial 16S rRNA gene sequence of strain Ka21^T^ was compared with the EzTaxon EzBioCloud Database (http://www.ezbiocloud.net/taxonomy) (Kim et al. 2012) for an approximate phylogenetic affiliation. After Sanger sequencing of the 16S rRNA gene, a genome sequencing project of Ka21^T^ was carried out, which revealed that there is only one 16S rRNA gene copy in the genome. Phylogenetic tree based on 16S rRNA gene was inferred by using the Maximum Likelihood method and Kimura 2-parameter model (Kimura 1980). The tree with the highest log likelihood (− 18,693.12) is shown in Fig. 3. Initial trees for the heuristic search were obtained automatically by applying Neighbor-Join and BioNJ algorithms to a matrix of pairwise distances estimated using the Maximum Composite Likelihood (MCL) approach, and then selecting the topology with superior log likelihood value. There were a total of 1491 positions in the final dataset. Evolutionary analyses were conducted in MEGA X (Kumar et al. 2018). For phylogenomic studies TYGS (https://tygs.dsmz.de/) (Meier-Kolthoff and Göker 2019), MiGA (http://microbial-genomes.org/) (Rodriguez et al. 2018) and GGDC (http://ggdc.dsmz.de/) (Meier-Kolthoff et al. 2013) webservers were used.

## Results and discussion

### Phenotypic and biochemical characterisation

LB medium was used for general laboratory cultivation, but the novel strain also grows well on TSA, nutrient and R2A media. After 72 h growth on LB agar at 30 °C, colonies were observed to be 1.0–1.5 mm in diameter, circular, non-mucoid, smooth and yellow. Strain Ka21^T^ was found to be Gram-reaction-negative, oxidase and catalase positive aerobic bacterium. Cells are non-motile, grow in 0.0–10.0% (w/v) NaCl, at a pH range from 6.5 to 9.0 and at temperatures between 10 and 35 °C. Optimal growth was observed at 30 °C, 1% (w/v) NaCl and pH 8.0. Cells of Ka21^T^ are short rods, the mean cell size is 0.5–0.7 µm in width and 1.5–2.0 µm in length (Online resource 1). According to API 50 CH test, Ka21^T^ produces acid from l-arabinose, d-xylose, d-glucose, d-mannose, esculin, and d-trehalose. Assimilation of d-glucose, l-arabinose, d-mannose, N-Acetyl-Glucosamine and d-maltose, hydrolysis of esculin and β-galactosidase activity were demonstrated by using the API 20 NE test. In the API ZYM test, strain Ka21^T^ showed activities of alkaline phosphatase, esterase lipase (C8), leucine arylamidase, naphthol-AS-BI-phosphohydrolase, β-glucosidase α-glucosidase, and N-acetyl-β-glucosaminidase. Distinctive physiological and biochemical characteristics of the isolate are given in Table 1.

**Table 1:** Differential characteristics of Ka21^T^ and the closely related strains

	1	2^a^	3^a^	4
Isolation source	Soil	Soil	Soil	Compost
Temperature range for growth (° C) (optimum)	10–35 (30)	10–35 (30)	10–40 (30)	10–45 (40)
Growth with NaCl (optimum) (%)	0–10 (1)	0–5 (1)	0–6 (1)	0–6 (0)
pH range for growth (optimum)	6.5–9.0 (8.0)	6.0–10.0 (7.0)	6.0–10.0 (8.0)	6.0–9.0 (8.0)
Oxidase activity	+	+	−	+
*Utilisation of*				
l-arabinose	+	−	+	+
d-mannose	+	−	+	+
d-mannitol	−	−	+	−
Capric acid	−	+	−	−
Trisodium citrate	−	+	−	−
*Activity of*				
Urease	−	−	+	−
Valine arylamidase	−	+	−	+
Cystine arylamidase	−	+	−	−
*α*-Chymotrypsin	−	−	+	−
*β*-Galactosidase	−	−	+	−
*α*-Mannosidase	−	−	+	−
esterase lipase (C8)	+	−	+	+
DNA G + C content (mol%)	41.0	40.6	36.0	42.3^a^

Strains: 1, Ka21^T^; 2, *Sphingobacterium olei* HAL-9^T^ (Liu et al. 2020); 3, *Sphingobacterium alkalisoli* Y3L14^T^ (Xu et al. 2017); 4, *Sphingobacterium composti* DSM 18850^T^ (Yoo et al. 2007)

^a^Data are from Liu et al. (2020) and Yoo et al. (2007) for the G + C content of *Sphingobacterium composti* DSM 18850^T^

### Chemotaxonomic characteristics

The predominant cellular fatty acids of strain Ka21^T^ are summed feature 3 (C_16:1_ *ω*7*c*/C_16:1_ *ω*6*c*, 33.6%), iso-C_15:0_ (32.5%) and iso-C_17:0_ 3OH (20.5%). The fatty acid profile is similar to that of related strains, in accordance with the description of *Sphingobacterium* genus (Steyn et al. 1998). However, the ratios of the different components are different. The complete fatty acid composition is shown in Table 2. The only respiratory quinone of Ka21^T^ is menaquinone-7 (MK-7). Strain Ka21^T^ exhibits a complex polar lipid profile consisting of phosphatidylethanolamine (PE) and phosphoglycolipid (PGL) as dominant elements, one aminoglycolipid (GNL), six phospholipids (PL) and six uncharacterised lipids (L) (Online resource 2).

**Table 2:** Cellular fatty acid composition of Ka21^T^ and related *Sphingobacterium* strains

	1	2	3^a^	4^a^
C_14:0_	tr	tr	1.2	tr
iso-C_15:0_	32.5	28.3	31.9	44.3
iso-C_15:0_ 3OH	2.3	2.2	2.3	1.9
anteiso-C_15:0_	tr	–	tr	tr
C_16:0_	1.2	4.7	4.4	3.1
C_16:0_ 3OH	tr	tr	2.2	tr
C_18:1_ *ω*9*c*	–	–	1.3	1.5
iso-C_17:0_ 3OH	20.5	17.6	13.7	17.3
Summed feature 3 (C_16:1_ *ω*7*c*/C_16:1_ *ω*6*c*)	33.6	40.1	38.9	25.6
Summed feature 9 (iso-C_17:1_ *ω*9*c/*C_16:0_ 10-methyl)	3.4	1.4	tr	1.6

Strains: 1, Ka21^T^; 2, *Sphingobacterium composti* DSM 18850^T^ (Yoo et al. 2007); 3, *Sphingobacterium olei* HAL-9^T^ (Liu et al. 2020); 4, *Sphingobacterium alkalisoli* Y3L14^T^ (Xu et al. 2017). tr, trace amount (< 1%); –, not detected

^a^Data are from Liu et al. (2020)

### Whole genome sequence analysis

The completeness and contamination values of the genome are 97.2% and 0.9%, respectively. Other quality labels of genome sequencing and assembly are as follows: 270-fold genome coverage, contig N50 = 433,102, number of contigs are 27. The genome size and G + C content of Ka21^T^ are 5,205,271 bp and 41.0 mol%, respectively. According to the annotation, there are 4320 genes, 4260 CDSs and 60 RNA genes in the genome. The coding density is 89.33%.

The RAST analysis revealed the presence of 242 subsystems, the subsystem coverage was 20% (Online resource 3).

The genome of Ka21^T^ contains 5 putative biosynthetic gene clusters (terpene, furan, arylpolyene, resorcinol, and type III polyketide synthase) in 4 genomic regions.

**Fig. 1:**
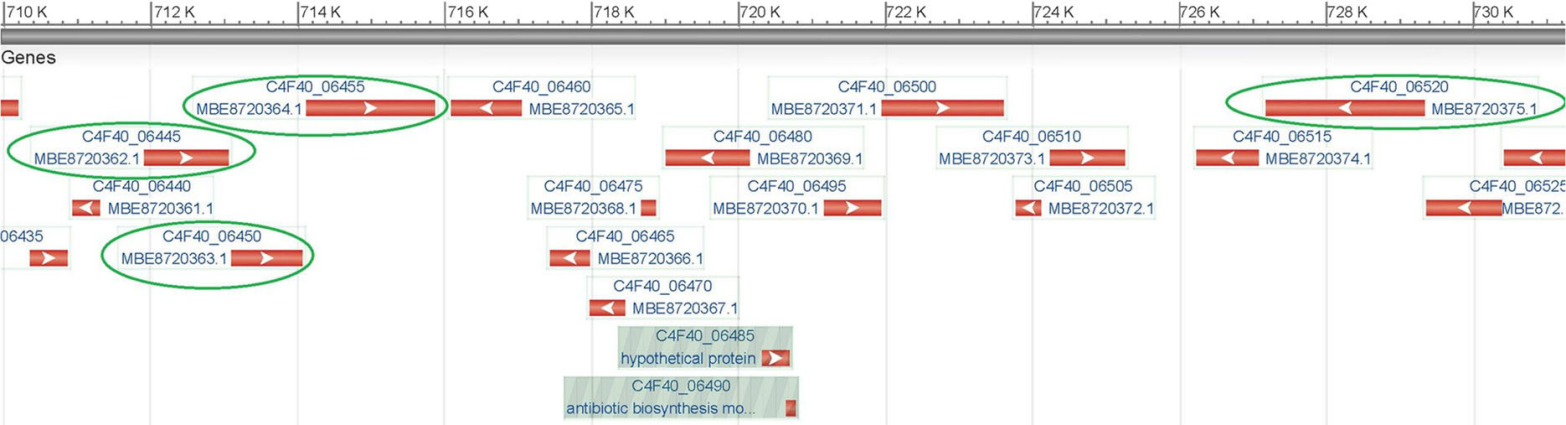
Island-like topology of four glycoside hydrolase genes (MBE8720362: 1,4-beta-xylanase; MBE8720363 and MBE8720364: alpha-N-arabinofuranosidase, MBE8720375: endo-1,4-beta-xylanase) involved in xylan degradation. The figure shows the region of contig 15 from 710 to 740 kbp. The arrows indicate the direction of genes. The codes above the arrows indicate the locus tags

Using the genome annotation and the Pfam database (Mistry et al. 2020; http://pfam.xfam.org/), several glycoside hydrolase (GH) genes in various GH families were found. These enzyme genes may play a role in the breakdown and modification of carbohydrates in soil. Some of the enzymes (in parentheses are the GenBank accession numbers) belonging to the GH1 (MBE8721502), GH2 (MBE8721947, MBE8722219, MBE8722220, MBE8720458), GH3 (MBE8720457, MBE8722817), GH9 (MBE8722726), GH10 (MBE8720362, MBE8720375, MBE8722731), GH16 (MBE8722218), GH26 (MBE8722637), GH43 (MBE8721705, MBE8721946, MBE8722218, MBE8721706, MBE8720364, MBE8720363) and GH130 (MBE8722632) families are active on plant cell wall polysaccharides (http://www.cazy.org/) (Lombard et al. 2014). These hydrolase genes are located on six contigs (number 15, 19, 20, 21, 22, 23) in the genome of Ka21^T^ and in the most cases, an island-like arrangement was found. Due to the complex structure of plant cell wall, its deconstruction needs collective work of several enzymes. Coordinated regulation of closely located genes may result in more efficient degradation. For example, on the contig 15, a region containing four GH genes (MBE8720362: 1,4-beta-xylanase; MBE8720363 and MBE8720364: alpha-N-arabinofuranosidase, MBE8720375: endo-1,4-beta-xylanase) that potentially play a role in xylan degradation was identified (Fig. 1).

**Fig. 2:**
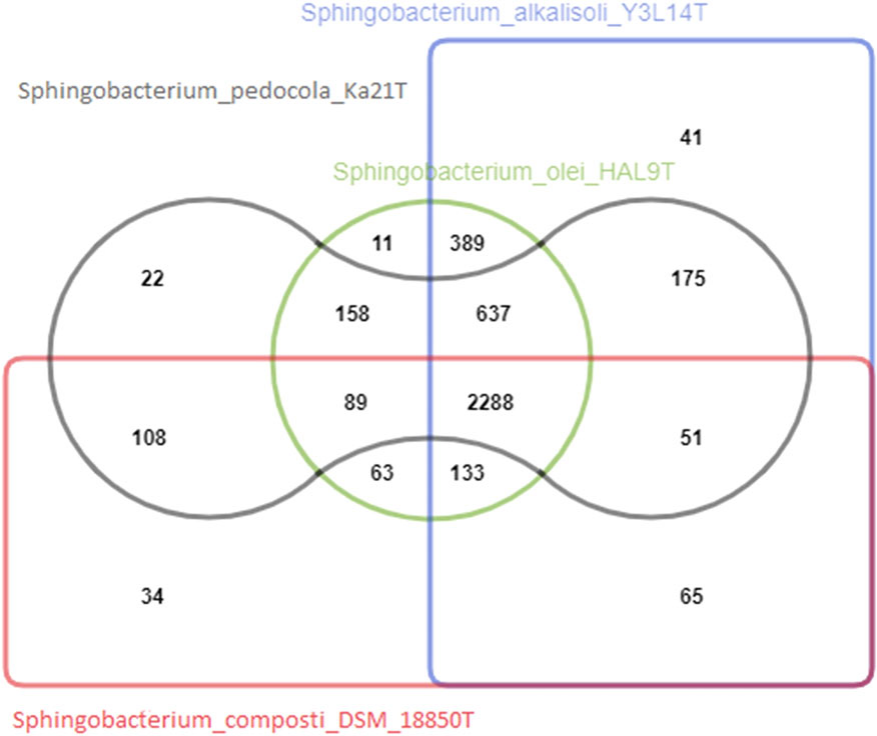
Comparative genome analysis for *Sphingobacterium pedocola* Ka21^T^, *Sphingobacterium alkalisoli* Y3L14^T^, *Sphingobacterium olei* HAL-9^T^ and *Sphingobacterium composti* DSM 18850^T^ was performed by OrthoVenn2 webserver. The numbers in the Venn-Diagram represent the number of clusters shared between strains. OrthoVenn2 generates clusters of proteins where each cluster consists of orthologs or paralogs from species. The overlapping cluster means the cluster contains proteins from different species

The comparative genome analysis for *Sphingobacterium pedocola* Ka21^T^, *Sphingobacterium alkalisoli* Y3L14^T^, *Sphingobacterium olei* HAL-9^T^ and *Sphingobacterium composti* DSM 18850^T^ revealed that the strains form 4264 clusters, 2051 orthologous clusters (at least contains two species) and 2213 single-copy gene clusters (Fig. 2). Methabolic pathway analysis revealed the elements of ppGpp biosynthesis (pathway completion value is 0.83), which nucleotide plays key role in the stress signalling system referred to as stringent response (Irving et al. 2021).

### Phylogenetic analysis

**Fig. 3:**
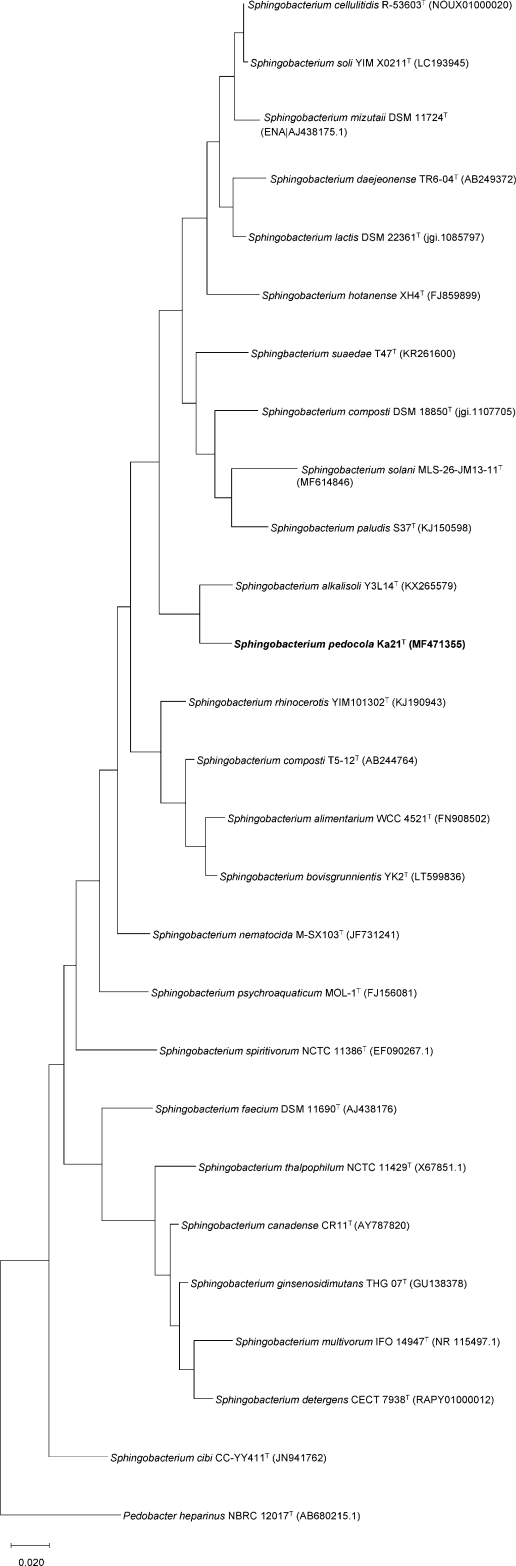
Maximum-likelihood tree based on 16S rRNA gene sequences showing the phylogenetic relationships between strain Ka21^T^ and related taxa

According to the comparisons with the partial (1343 bp) 16S rRNA gene sequences in the EzTaxon database, highest level of sequence similarity occurred with *Sphingobacterium alkalisoli* Y3L14^T^ (96.72%) (Xu et al. 2017), *Sphingobacterium olei* HAL-9^T^ (96.35%) (Liu et al. 2020) and *Sphingobacterium composti* DSM 18850^T^ (95.23%) (Yoo et al. 2007). The 16S rRNA gene based phylogeny tree suggests that strain Ka21^T^ forms a distinct phyletic lineage within *Sphingobacterium* genus (Fig. 3).

According to genome based analysis, the closely related taxons found by MiGA are *Sphingobacterium olei* HAL-9^**T**^ (GenBank assembly accession: GCA_005048855) (86.5% ANI) and *Sphingobacterium alkalisoli* Y3L14^T^ (GenBank assembly accession: GCA_005049105**)** (84.39% ANI). The p-value of taxonomic novelty at species level is 0.00292.

**Fig. 4:**
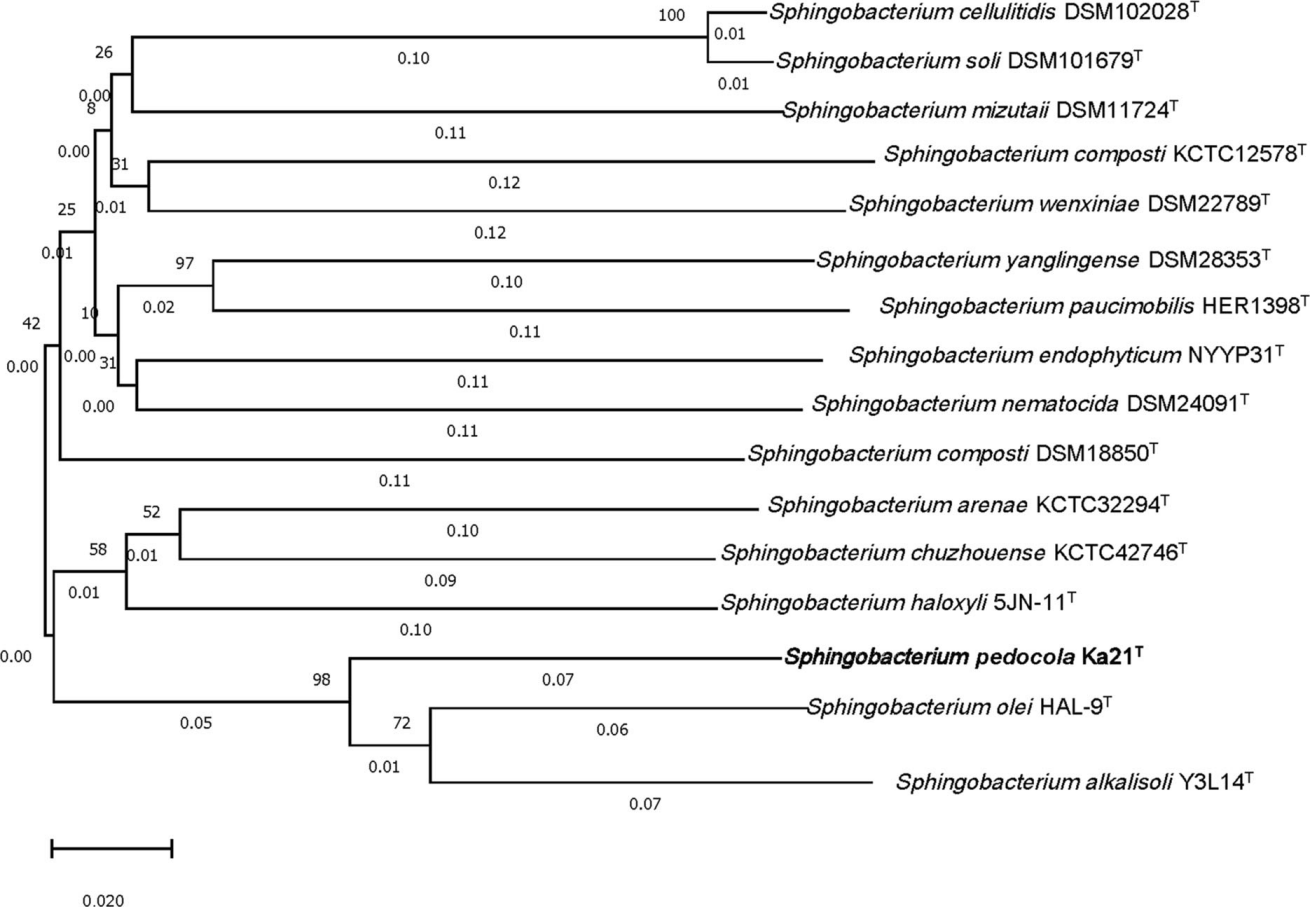
Tree inferred with FastME 2.1.6.1 (Lefort et al. 2015) from GBDP distances calculated from genome sequences. The branch lengths are scaled in terms of GBDP distance formula _δ_5. The numbers above branches are GBDP pseudo-bootstrap support values from 100 replications, with an average branch support of 39.9%. The tree was rooted at the midpoint (Farris 1972)

dDDH values (identities/HSP length) between Ka21^T^ and *Sphingobacterium olei* HAL-9^**T**^ and Ka21^T^ and *Sphingobacterium alkalisoli* Y3L14^T^ are 31.60% and 28.20%, respectively. Whole genome based tree generated by TYGS also confirmed the taxonomic position of Ka21^T^ within *Sphingobacterium* genus as a novel species (Fig. 4).

According to the 16S rRNA based and whole genome based phylogenetic analyses, Ka21^T^ represents a novel species in genus *Sphingobacterium*. The generally accepted species boundary for 16S rRNA gene similarity, ANI and dDDH values are 98.7%, 95–96% and 70%, respectively (Meier-Kolthoff et al. 2013; Chun et al. 2018; Stackebrandt and Ebers 2006; Goris et al. 2007; Richter and Rosselló-Móra 2009). Obtained values for Ka21^T^ (96.72% for 16S rRNA gene similarity, 86.5% for ANI and 31.6% for dDDH) are all lower, confirming the results of phylogenetic treeing.

## Protologue

In conclusion, the revealed characteristics of Ka21^T^ exhibit the typical traits of the genus *Sphingobacterium*: Gram-negative, non-motile and rod shaped cells, positive for catalase and oxidase, MK-7 as respiratory quinone, major amount of phosphatidylethanolamine in the polar lipid profile, fatty acid profile with C_16:1_ *ω*7*c*/C_16:1_ *ω*6*c*, iso-C_15:0_, iso-C_17:0_ 3OH as the most dominant compounds and low genomic G + C content. According to 16S rRNA gene and whole genome based phylogenetic trees, strain Ka21^T^ occupies a separate lineage in the genus. The 16S rRNA gene sequence similarities to the closely related taxons and overall genome related indices (ANI, dDDH) also indicate its distance from other species. Phenotypic, biochemical, chemotaxonomic and phylogenetic information of strain Ka21^T^ support its classification as a novel species of *Sphingobacterium*, for which the name *Sphingobacterium pedocola* sp. nov. is proposed. The GenBank accession numbers for the 16S rRNA gene sequence and the whole genome of *Sphingobacterium pedocola* strain Ka21^T^ are MF471355 and PSKQ00000000, respectively.

## Description of *Sphingobacterium pedocola* sp. nov.

*Sphingobacterium pedocola* (pe.do′co.la. Gr. neut. n. *pedon* soil; L. suff. *-cola* inhabiting; N.L. n. *pedocola* soil-inhabiting). Cells are strictly aerobic, Gram-reaction-negative straight rods and non-motile. It grows well on TSA, LB, nutrient and R2A plates. Colonies have yellow pigmentation on LB after 72 h incubation. Cells are 0.5–0.7 µm in width and 1.5–2.0 µm in length. It grows at 10–35 °C (optimum, 30 °C) and at NaCl concentrations of 0.0–10.0 w/v % (optimum, 1w/v %). The major fatty acids are summed feature 3 (C_16:1_ ω7c/C_16:1_ *ω*6*c*), iso-C_15:0_ and iso-C_17:0_ 3OH. The only respiratory quinone is MK-7. The major polar lipids are phosphatidylethanolamine and phosphoglycolipid. The DNA G + C content of the type strain is 41.0 mol%. The type strain is Ka21^T^ (= LMG 31575 = NCAIM B.02636) isolated from agricultural field in the Great Hungarian Plain, Hungary.

## Supplementary Information

Below is the link to the electronic supplementary material.

Supplementary file1 (PDF 375 KB)

Supplementary file2 (PDF 309 KB)

Supplementary file3 (PDF 318 KB)

## Data Availability

The GenBank accession numbers for the 16S rRNA gene sequence and the whole genome of *Sphingobacterium pedocola* strain Ka21^T^ are MF471355 and PSKQ00000000, respectively.

## Abbreviations

ANI: Average Nucleotide Identity
dDDH: digital DNA–DNA hybridisation
DSMZ: Deutsche Sammlung von Mikroorganismen und Zellkulturen (German Collection of Microorganisms and Cell Cultures)
GGDC: Genome-to-Genome Distance Calculator
GNL: Aminoglycolipid
L: Lipid
LB agar: Luria–Bertani agar
MiGA: Microbial Genomes Atlas
MLSA: MultiLocus Sequence Analysis
PE: Phosphatidylethanolamine
PGL: Phosphoglycolipid
PL: Phospholipid
RAST: Rapid Annotation using Subsystem Technology
TYGS: Type Strain Genome Server
