# Supplementary material for: Sphingobacterium pedocola sp. nov. a novel halotolerant bacterium isolated from agricultural soil
Source: Antonie Van Leeuwenhoek. 2021 Aug 6;114(10):1575–84. doi: 10.1007/s10482-021-01623-6 (PMC8448689; doi:10.1007/s10482-021-01623-6)

Sphingobacterium pedocola sp. nov. a novel halotolerant bacterium isolated from agricultural soil

Antonie van Leeuwenhoek

Ákos Tóth[1*], Ildikó Bata-Vidács[1], Judit Kosztik[1], Rózsa Máté[2], József Kutasi[2], Erika Tóth[3], Károly Bóka[4], András Táncsics[5], István Nagy[6], Gábor Kovács[6,7], József Kukolya[1]

[*]Correspondence: Tóth Á; affiliation: Research Group for Food Biotechnology, Institute of Food Science and Technology, Hungarian University of Agriculture and Life Sciences, Budapest, Hungary; e-mail address: toth.akosgergely@gmail.com

Supplementary figures 3. Subsystems of strain Ka21^T revealed by RAST server version 2.0.

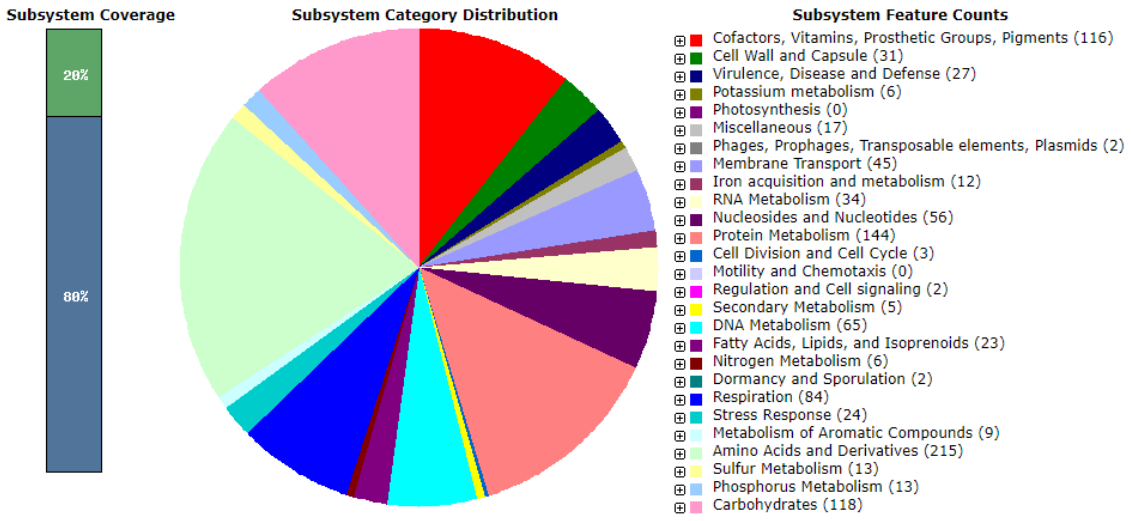

Supplement: Supplementary file 3 — Supplementary file3 (PDF 318 KB) [file 10482_2021_1623_MOESM3_ESM.pdf]
